# Chinese herbal injections for radiation pneumonitis: A protocol for systematic review and meta-analysis

**DOI:** 10.1097/MD.0000000000028929

**Published:** 2022-02-25

**Authors:** Yuerong Gui, Qing Pang, Shuo Wang, Jun Dong, Dandan Wang, Xiumei Ma, Xinyan Wang, Shuaihang Hu, Wei Hou

**Affiliations:** a Guang’an men Hospital, China Academy of Chinese Medical Sciences, China; b Beijing University of Chinese Medicine, China.

**Keywords:** Chinese herbal injections, efficacy, radiation pneumonitis, randomized controlled trial, systematic review

## Abstract

**Background:**

Radiation pneumonitis is a common dose-limiting factor in radiotherapy for thoracic malignancies, and its treatment encounters a bottleneck. As an essential adjuvant treatment method, Chinese herbal injections (CHIs) have been used to treat radiation pneumonitis (RP), and clinical studies have appeared potentially beneficial and nontoxic. However, the efficacy and safety of CHIs for RP have not been evaluated comprehensively.

**Methods:**

The systematic review and meta-analysis will be performed according to the Preferred Reporting Items for Systematic Reviews and Meta-Analysis Protocol (PRISMA-P) statement guidelines. The Cochrane Library, PubMed, EMBASE, SinoMed, CNKI, VIP, and Wan Fang Databases were systematically searched from inception until January 20, 2022. The selection of studies, data extraction, and assessment of the risk of bias will be performed by 2 reviewers independently. The total effective rate was used as a primary outcome measure; the secondary outcomes are quality of life, clinical symptoms and signs, inflammatory cytokines, and adverse effects. Cochrane Review Manager (RevMan5.3) software will be used for data synthesis and analysis.

**Results and conclusion:**

This systematic review will evaluate the efficacy and safety of CHIs in treating radiation pneumonitis to provide more comprehensive evidence for the treatment of clinical RP.

**INPLASY registration number:**

INPLASY202210106.

## 1 Introduction

Radiation therapy (RT) remains a cornerstone of treatment in definitive (i.e., curative) and palliative settings for many malignancies.^[1]^ However, as we know, the lungs are exquisitely sensitive to the deleterious effects of ionizing radiation.^[2]^ Radiation pneumonitis (RP) is a common toxicity after RT or stereotactic body radiotherapy for thoracic malignant tumors, usually occurring within 6 months of therapy.^[1]^ Research shows RP affects between 15% and 40% of people who undergo RT for lung cancer.^[3]^ It may also develop in patients who undergo chest radiation for breast cancer (1%–5%), mediastinal lymphomas (5%–10%), thymic tumors, or esophageal cancer.^[4,5]^ The significant consequence of RP is shortness of breath, which leads to diminished quality of life (QoL) and reduced activities of daily living. By 12 to 24 months, most patients receiving moderate-high doses of RT have radio-logic evidence of lung fibrosis, which is the permanent scarring of lung tissue and leads to permanent impairment of oxygen transfer.^[5,6]^ This condition can make breathing very difficult and even lead to death. Thus, partial remission (PR) is an essential dose-limiting factor among patients receiving thoracic RT. So far, RP treatment is aimed at decreasing inflammation. Glucocorticoids are the main course of treatment.^[2]^ Unfortunately, glucocorticoids can produce many adverse reactions during use, such as hyperglycemia, dyslipidemia, cardiovascular disease, osteoporosis, etc.^[7]^ Based on the above limitations, its use is limited in clinical practice.

As an important adjuvant therapy for RP, traditional Chinese medicine (TCM) has gained widespread clinical applications in China and other parts of Asia. A meta-analysis that included 56 clinical trials showed that Chinese herbal medicine could significantly improve the symptoms and imaging manifestations of patients with RP and appeared potentially beneficial and nontoxic.^[8]^ Chinese herbal injections (CHIs) are a new dosage form of TCM based on more modern technologies, which have the advantages of production line standard, high bioavailability, and fast onset of action and play an essential role in the medical system of China.^[9]^ Many studies indicated that CHIs had apparent advantages in preventing the occurrence of RP, reducing the degree of inflammation, improving symptoms, enhancing the QoL, improving tolerance to cortisol treatment, and reducing adverse reactions. For example, in patients with radiation therapies combined with Compound Kushen Injection for 2 weeks, the incidence rate of radiation lung injury was significantly lower than that of patients with simple radiotherapy (36.70% vs 64.66%)^[10]^; after 14 days of glucocorticoids plus antibiotics combined with Xue Bi Jing Injection, the effective rate was significantly higher than that of the patients with glucocorticoids plus antibiotics alone (85.7% vs 57.6%), according to International Union Against Cancer RP efficacy judgment criteria.^[11]^ However, few systematic reviews of CHIs in the treatment of RP have been published, especially for all types of CHIs. Systematic reviews, usually provide the highest level of evidence for a clinical problem by systematically reviewing and meta-analyzing the results of multiple randomized controlled trials (RCTs) addressing the same clinical problem to guide clinical practice.^[12]^ Therefore, this study conducted an SR of RP treatment with CHIs to provide an evidence reference for the clinical application of CHIs.

## 2 Method

### 2.1 Study registration

This systematic review has been registered on INPLASY as INPLASY202210106 (https://inplasy.com/inplasy-2022-1-0106/) and will be performed according to the Preferred Reporting Items for Systematic Reviews and Meta-Analysis Protocol (PRISMA-P) statement guidelines.^[13,14]^ Ethical approval was not required for this study as all the research materials are derived from published studies.

### 2.2 Inclusion criteria

#### 2.2.1 Types of studies

RCTs that investigated the effect of CHIs for the treatment of RP will be included. No language limitation exists.

#### 2.2.2 Participants

Patients (aged 18 years or older) with RP, which should be confirmed according to the clinical diagnostic standard.^[2,15]^ According to the severity of clinical symptoms, the patients were divided into grades 0 to 5 according to the Radiation Therapy Oncology Group (RTOG) standard, RP ≥ grade 2, and patients requiring active treatment, the course of the disease was less than 6 months. There are no limitations in gender, education, race, or nationality.

#### 2.2.3 Interventions

Control group: placebo/no intervention, or conventional treatment such as Glucocorticoid and/or antibiotics;

Test group: Based on the control group, use CHIs intravenously. There is no limitation on the dose and intervention time of CHIs.

In addition to the above treatment, both groups can receive the same basic treatment, such as oxygen absorption, cough clearance, phlegm reduction, and bronchiectasis. For specific treatment methods, refer to the Expert consensus for the Diagnosis and treatment of radiation-induced lung injury.^[15]^

#### 2.2.4 Outcomes

Primary outcomes:

Total effective rate: According to International Union Against Cancer criteria for judging the efficacy of RP,^[16]^ the pneumonia remissions were evaluated as complete remission, PR, and not cured.

The total effective rate refers to the proportion of patients with complete remission plus PR.

Secondary outcomes:

1. QoL: The patient's QoL was measured before and after treatment using the Karnofsky performance status scale. An increase of 10 points or more on the Karnofsky performance status score was considered an improvement.
2. Clinical symptoms and signs: Duration of fever, cough, asthma exacerbation, and colored sputum was evaluated.
3. Inflammatory Cytokines. Interleukin-6 (IL-6), TGF-β, and TNF-α cytokines in plasma were evaluated.
4. Incidence of adverse events: Adverse events related to CHIs intervention were extracted.

### 2.3 Exclusion criteria

1. Duplicated publications, non-clinical research, case reports, commentaries.
2. In addition to the CHIs studied, other TCM interventions were also used.
3. Studies with inadequate CHIs quality control will be excluded.
4. Documents of data errors.

### 2.4 Database and search strategies

The Cochrane Library, PubMed, EMBASE, SinoMed, CNKI, VIP, and Wan Fang Databases were systematically searched from inception until January 20, 2022. References about related systematic reviews in this research field were searched manually, and relevant experts in the field were consulted. The search strategy of PubMed is shown in Table 1.

**Table 1:** Search strategy for the PubMed database.

Number	Search items
#1	“Radiation Pneumonitis”[Mesh]
#2	Pneumonia, Radiation[tiab] OR Pneumonitis, Radiation[tiab] OR Radiation Pneumonia[tiab] OR Radiation Pneumonitis[tiab] OR Radiation-Induced Lung Injury[tiab] OR Radiation Induced Lung Injury[tiab] OR radiation induced pneumonitis[tiab] OR radiation induced pneumonia[tiab]
#3	#1 OR #2
#4	Chinese herbal injection[tiab] OR Chinese medicine injection[tiab] OR injection of TCM[tiab] OR Aidi[tiab] OR Ai Di[tiab] OR Compound Kushen[tiab] OR Danshen[tiab] OR Danshen Ligustrazine[tiab] OR Dan Xiang[tiab] OR Elemene[tiab] OR Lian Bi Zhi[tiab] OR Lianbizhi[tiab] OR Ligustrazine[tiab] OR Shen Fu[tiab] OR Shenfu[tiab] OR Shuang Huang Lian[tiab] OR Shuanghuanglian[tiab] OR Shen Mai[tiab] OR Shenmai[tiab] OR Shen Qi Fu Zheng[tiab] OR Shenqifuzheng[tiab] OR Tan Re Qing[tiab] OR Tanreqing[tiab] OR Xue Bi Jing[tiab] OR Xuebijing[tiab]
#5	#3 AND #4

### 2.5 Study selection

Import all retrieved documents into EndNote X9 software for management. Literature screening followed the PRISMA statement.^[17]^ Firstly, filter out duplicate documents; Then, 2 researchers will read the title and abstract of the article initially, filter out irrelevant documents, and finally read the full text of the article to filter out documents that do not meet the inclusion criteria. Any discrepancies will be resolved by mutual negotiation or be decided by the third reviewer.

### 2.6 Data extraction

After completing the literature screening, the two researchers performed information extraction independently. The information extraction included:

1. Study ID: first author names and publication year;
2. Patient information: median age, number of patients, gender;
3. Intervention: CHI names, dosages, duration of treatment;
4. Outcomes: the total effective rate, QoL, clinical symptoms and signs, inflammatory cytokines, and adverse drug events within the treatment.

Two researchers will independently extract data and check for consistency. In case of disagreement, it can be resolved through negotiation, or a third-party researcher can make a joint decision.

### 2.7 Risk of bias assessment

Two reviewers will assess the risk of bias of the included studies by the “Risk of Bias Assessment Tool” of the Cochrane Handbook for RCTs.^[18]^ The evaluation contents include random sequence generation, allocation concealment, blinding of participants and personnel, blinding of outcome assessment, incomplete outcome data, selective reporting, and other biases. Each item is divided into “high risk”, “unclear risk”, and “low risk”. Any inconsistencies will be determined in consultation with the third reviewer.

### 2.8 Data synthesis and analysis

#### 2.8.1 Data synthesis

Statistical analysis will be performed with Review Manager software 5.3. Relative risk is used to evaluate the effect size for binary variables, and the mean difference is used as the efficacy analysis statistic for continuous variables. Heterogeneity between results will be assessed by the value of *P* and *I*^2^, *P* > .1, and *I*^2^ < 50% indicate small heterogeneity; *P* < .1 and *I*^2^≥50% indicate high heterogeneity. The fixed-effect model was used inhomogeneity data merging, and the random-effects model was suitable for the merging of heterogeneous data. If there is clinical and methodological heterogeneity, subgroup analyses, meta-regression analyses, or descriptive analyses were performed as appropriate.

#### 2.8.2 Subgroup analysis

If the results to be analyzed are heterogeneous, analyze the source of the heterogeneity, and perform subgroup analysis according to the patient's age, Radiation Therapy Oncology Group classification, the type of CHIs, the duration of treatment, medication dosage, etc.

#### 2.8.3 Sensitivity analysis

If the risk of bias of the studies is high, sensitivity analysis will be performed to investigate the asymmetry of funnel plots to exclude low-quality studies. Evaluate whether the results of the meta-analysis are stable and reliable.

#### 2.8.4 Reporting bias

If the number of meta-analyses is ≥9,^[19]^ an inverted funnel plot will be drawn to judge the publication bias of the included studies. If the asymmetry exists in the funnel plots, the Egger tests will be performed to analyze potential causes.

### 2.9 Quality of evidence

Two researchers will separately evaluate the quality of the review based on the Grading of Recommendations Assessment Development and Evaluation.^[20]^ Any inconsistencies will be determined by a third researcher.

## 3 Discussion

RP is an important dose-limiting factor in cancer patients with thoracic radiation. One analysis determined that 2.3% of all patients who received thoracic radiotherapy or chemoradiotherapy for advanced cancer died of RP.^[21]^ But its treatment encounters a bottleneck. CHI, as a new dosage form of TCM, has the advantages of no gastrointestinal absorption and fast onset of action and has been widely used in the treatment of RP in China. Several clinical studies have shown that CHIs have good efficacy and safety in the treatment of RP.^[11,22–25]^ However, no SR of CHIs for RP has been published. Therefore, this study will carry out an SR of CHIs for RP to guide the clinical application of CHIs in the treatment of RP.

There are certain limitations to this study. Articles using CHIs as intervention measures are mostly published in China, and there is a possibility of regional bias; Second, there is the possibility that quantitative pooling cannot be performed due to the lack of included articles; finally, the poor methodological quality of the included literature may affect the degree of confidence in the effect estimates.

## Author contributions

**Conceptualization:** Wei Hou.

**Data curation:** Xiumei Ma, Xinyan Wang, Yuerong Gui.

**Investigation:** Yuerong Gui, Qing Pang.

**Methodology:** Shuaihang Hu, Yuerong Gui, Shuo Wang.

**Resources:** Yuerong Gui, Qing Pang.

**Supervision:** Jun Dong, Dandan Wang, Wei Hou.

**Writing – original draft:** Yuerong Gui, Qing Pang.

**Writing – review & editing:** Yuerong Gui, Qing Pang, Shuo Wang.
